# Combined X-ray Crystallographic, IR/Raman Spectroscopic, and Periodic DFT Investigations of New Multicomponent Crystalline Forms of Anthelmintic Drugs: A Case Study of Carbendazim Maleate

**DOI:** 10.3390/molecules25102386

**Published:** 2020-05-21

**Authors:** Alexander P. Voronin, Artem O. Surov, Andrei V. Churakov, Olga D. Parashchuk, Alexey A. Rykounov, Mikhail V. Vener

**Affiliations:** 1 Department of Physical Chemistry of Drugs, G.A. Krestov Institute of Solution Chemistry of RAS, 153045 Ivanovo, Russia; flox-av@yandex.ru (A.P.V.); aos@isc-ras.ru (A.O.S.); 2 Department of Crystal Chemistry and X-ray Diffraction, N.S. Kurnakov Institute of General and Inorganic Chemistry of RAS, 119991 Moscow, Russia; churakov@igic.ras.ru; 3 Faculty of Physics, Lomonosov Moscow State University, 119991 Moscow, Russia; olga_par@rambler.ru; 4 Theoretical Department, FSUE “RFNC-VNIITF Named after Academ. E.I. Zababakhin”, 456770 Snezhinsk, Russia; arykounov@gmail.com; 5 Department of Quantum Chemistry, D. Mendeleev University of Chemical Technology, 125047 Moscow, Russia

**Keywords:** conventional and non-conventional H-bonds, empirical Grimme corrections, lattice energy of organic salts, computation of low-frequency Raman spectra

## Abstract

Synthesis of multicomponent solid forms is an important method of modifying and fine-tuning the most critical physicochemical properties of drug compounds. The design of new multicomponent pharmaceutical materials requires reliable information about the supramolecular arrangement of molecules and detailed description of the intermolecular interactions in the crystal structure. It implies the use of a combination of different experimental and theoretical investigation methods. Organic salts present new challenges for those who develop theoretical approaches describing the structure, spectral properties, and lattice energy *E*_latt_. These crystals consist of closed-shell organic ions interacting through relatively strong hydrogen bonds, which leads to *E*_latt_ > 200 kJ/mol. Some technical problems that a user of periodic (solid-state) density functional theory (DFT) programs encounters when calculating the properties of these crystals still remain unsolved, for example, the influence of cell parameter optimization on the *E*_latt_ value, wave numbers, relative intensity of Raman-active vibrations in the low-frequency region, etc. In this work, various properties of a new two-component carbendazim maleate crystal were experimentally investigated, and the applicability of different DFT functionals and empirical Grimme corrections to the description of the obtained structural and spectroscopic properties was tested. Based on this, practical recommendations were developed for further theoretical studies of multicomponent organic pharmaceutical crystals.

## 1. Introduction

Organic salts are crystalline ionic compounds that contain one or more organic ions in their structure. Organic salts have broad application in the pharmaceutical industry [1], non-linear optics [2], catalysis [3], green solvents for chemical production [4], etc. Rational design of organic salts and relative materials implies the development of computational methods capable of reliable prediction of industry-relevant properties such as spectroscopic features and crystal lattice energy.

There are several benchmark sets of single-component organic crystals consisting of small rigid molecules without organic fluorine, packed together by van der Waals forces and/or weak and moderate hydrogen bonds (H-bonds), whose lattice energy is accurately computed [5,6,7,8,9]. New and existing theoretical methods are developed and tested based on these sets, and then they are further applied to various compounds. However, most crystals with actual or potential practical application have little in common with the structures from the benchmark sets. Some examples include single-component crystals of larger, conformationally flexible molecules [10,11,12,13,14], fluoroorganic compounds [15,16], and multicomponent crystals [17,18,19], often with short (strong) [20,21] or ionic H-bonds [22]. The applicability of the methods tested against the benchmark sets for modeling the properties of non-model crystals (e.g., organic salts) is unclear.

In order to describe the properties of “real” crystals, semi-empirical methods based on additive schemes and/or parameterized force fields are often used [23,24,25]. Their area of application is often limited to a single property (usually to crystal lattice energy), and they are unable to describe a number of properties determined experimentally, including IR and Raman spectra, electron density distribution, etc. The semi-empirical methods provide accurate values of sublimation enthalpies of one-component crystals, consisting of molecules of an arbitrary size [26] and two-component crystals with non-conventional H-bonds [27]. However, these methods are very sensitive to force field parameterization.

For this reason, we chose periodic density functional theory (DFT) methods which allow describing a wide range of properties of the crystalline phase, and which have relatively low computational costs even when treating complex multicomponent crystals [28] of large flexible molecules [29,30] containing aromatic fluorine [31], as well as short (strong) or ionic H-bonds [32,33]. We believe that periodic (solid-state) DFT computations provide a grounded trade-off between the accuracy and the rate of calculations of experimentally observed properties of multi-component organic crystals. In the DFT methods, there are two main approaches based either on Gaussian-type orbitals (GTO) or on plane waves (PW), both with their advantages and disadvantages. Thus, GTO basis sets can better describe isolated molecules in the gas phase, which is essential for *E_latt_* calculation [33], while many solid-state properties such as solid-state infrared (IR) spectra are traditionally computed using PW [34]. Only a few articles provide a comparison of the results obtained with GTO and PW bases [35,36]. The choice of the functional is also important for the quality of the obtained data. For example, the B3LYP (Becke 3-parameter, Lee-Yang-Parr) functional is commonly applied in computations with GTOs [37,38], while PBE (Perdew-Burke-Ernzerhof) is used with PW basis sets [30,34,39]. It is well known that B3LYP describes systems with short (strong) or ionic H-bonds better than PBE, while the latter overestimates the stabilization energy in molecular complexes and crystals [40,41]. Non-directed dispersion interactions cause problems for DFT computations in both the GTO and PW versions, making it necessary to use the dispersion corrections of different nature. However, it is not yet investigated how the dispersion corrections [42,43,44], as well as other parameters such as optimization of cell parameters [7,9,30,34], type of the functional, and basis set, affect the observable properties (e.g., sublimation enthalpy [28,45], IR/Raman spectra [46,47,48], metric [18,22] and electron density features [42] at bond critical points of conventional and non-conventional hydrogen bonds) of molecular crystals with short (strong) or ionic H-bonds.

Some technical problems that a user of periodic (solid-state) DFT programs encounters when calculating the properties of multicomponent organic crystals remain unsolved. It is still unclear how full optimization (variation of cell parameters) influences the metric parameters of short/ionic H-bonds, *E_latt_* values, the wave numbers of normal vibrations in the low-frequency region <400 cm^−1^, etc.

Soluble drug forms are one of the main areas of application of multicomponent crystals. For this reason, anthelmintic compounds with low aqueous solubility were selected as objects of the present study. Anthelmintic benzimidazole derivatives are basic compounds capable of forming a variety of two-component crystals with pharmaceutically acceptable acids [49,50,51]. Since the number of potential pharmaceutical crystal forms of target compounds is very high, accurate theoretical estimates of relevant properties are desired to avoid excessive experimental work.

**Figure 1:**
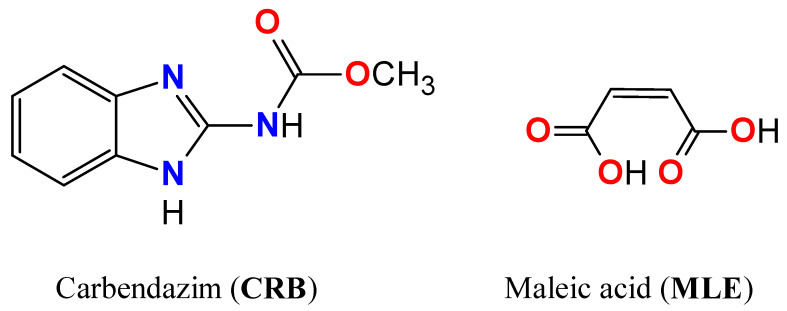
Molecular structures of carbendazim (**CRB**) and maleic acid (**MLE**).

In this paper, the new two-component crystalline form of the anthelmintic drug 1:1 carbendazim–maleic acid crystal [CRB + MLE] (1:1) is investigated using X-ray and IR/Raman spectroscopy in combination with periodic (solid-state) DFT calculations (Figure 1). The applicability of different DFT functionals and empirical Grimme corrections to reproducing the experimentally observed parameters was tested using the Crystal17 and QuantumEspresso DFT codes. As a result, “practical recipes” are proposed for computing multicomponent organic crystals for users of these programs.

## 2. Results and Discussion

The crystallographic data of the [CRB + MLE] (1:1) salt were recorded at 120 K and at room temperature to study the temperature effect on the metric parameters of the unit cell and most notable H-bonds. The crystallographic information is collected in Table S1 (Supplementary Materials). We see that the thermal expansion in the interval between 120 K and 296 K is almost negligible, as the cell volume increases only by 3% (44 Å^3^).

The asymmetric unit contains one CRB cation and one MLE anion. The crystal has H-bonds of different types and strengths: conventional intra- and intermolecular H-bonds and non-conventional C–H···O contacts. A number of equations were proposed to assess the dependence of the H-bond stabilization energy from the distances between the heavy atoms [52,53], H···O/N distance [54], and electron density descriptors [41,42]. According to these approaches, the intramolecular O24–H24···O21 bond can be considered strong, while the two intermolecular N–H···O bonds can be classified as medium or weak hydrogen bonds (Table S2, Supplementary Materials).

### 2.1. Hydrogen Bond Patterns

**Figure 2:**
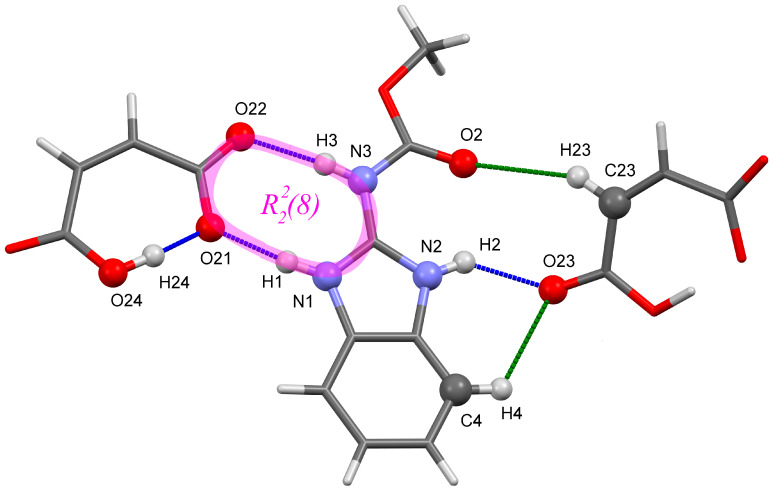
Part of the hydrogen bond network in [CRB + MLE] (1:1). The H-bonds and C–H···O contacts are colored blue and green, respectively.

The molecules in the asymmetric unit are combined into a heterodimer formed by ionic N^+^1–H1···O21 and medium or weak N3–H3···O22 bonds, which build the eight-membered cyclic motif with the $R_{2}^{2}(8)$ graph set notation [55]. Another conventional N2–H2···O23 hydrogen bond connects the N–H group of the CRB cation with the adjacent MLE anion and is assisted by two C–H···O contacts (Figure S1, Supplementary Materials). The O21 atom acts as an acceptor of two H-bonds, short (strong) intramolecular and ionic intermolecular ones (Figure 2).

### 2.2. Effect of Optimization on Cell Parameters

The volume of the crystallographic cell of the considered two-component crystal increases from 1400.5 to 1444.6 Å^3^ as the temperature rises from 120 to 296 K. This means that the cell parameters change slightly when the temperature increases. This result is consistent with the published data [56], according to which the thermal expansion from 120 to 296 K for organic crystals is estimated to range between ~1% and ~3%. The sign and absolute value of the relative change in the volume of the crystallographic cell depend on the functional and type of Grimme corrections (Table 1). The data given in this table indicate that none of the approximations reproduce the experimental value of the thermal expansion of the considered crystal. Some approximations give negative values of this coefficient. Note that such a result was already obtained in a number of articles (see Tables 2 and 3 in Reference [57], Table 2 in Reference [58], Figure 4 in Reference [59], and Table S19 in Reference [34]). These results demonstrate that the change in the unit cell volume during the lattice optimization does not always correspond to the experimental data.

### 2.3. Metric Parameters of Conventional and Non-Conventional H-Bonds

The experimental values of the distances between the heavy atoms involved in the formation of conventional H-bonds are compared in Table 1 with the theoretical values computed using different levels of approximation with fixed unit cell parameters and full unit cell relaxation. We assume that the calculations are in agreement with the X-ray data if the theoretical values of the O···O and O···N distances differ from the experimental ones by no more than 0.01 Å. The data presented in Table 1 suggest that (i) the considered distances are very sensitive to the choice of the functional (B3LYP or PBE), the inclusion of the dispersion correction, and its type (D2, D3, or none), (ii) the variation of the cell parameters greatly changes the distances if the relative change in the volume of the crystallographic cell is more than a few percent, and (iii) the results we get also depend on the basis set type (PW or GTO).

The metric parameters of the non-conventional H-bonds extracted from the experimental crystal structure are poorly reproduced by all the approximations used (Table S3, Supplementary Materials). We can draw two conclusions. Firstly, the B3LYP approximation with the fixed cell parameters gives the best description of the metric parameters of the conventional H-bonds in the considered crystal. Secondly, it is almost impossible to describe the distances between the heavy atoms involved in the formation of the non-conventional H bonds with an accuracy of ~0.01 Å in the framework of the approximations used.

### 2.4. IR Spectrum in the Low-Frequency Region

The IR spectrum of [CRB + MLE] (1:1) can be divided into high-frequency (>1800 cm^−1^), low-frequency (<400 cm^−1^), and mid-frequency spectral ranges (Figures S2 and S3, Supplementary Materials). For a correct description of the IR frequencies of the asymmetric vibrations of the O–H···O and O–H···N/^−^O···H–N^+^ fragments in the high-frequency range, it is necessary to go beyond the double harmonic approximation framework [60]. Explicit accounting of mechanical and electric anharmonicity is very cumbersome and time-consuming [61], especially in the case of organic crystals with intermolecular H-bonds [62,63]. The mid-frequency spectral range is usually described well in the cluster approximation; in some cases, the cluster can consist of one molecule [64].

We focus on reproducing the frequencies, as well as IR and Raman activities, in the low-frequency spectral range. It is currently being intensively studied [29,65,66], since various intermolecular vibrations can be observed in it, in particular, because of the presence of intermolecular H-bonds [36,67,68,69]. The double harmonic approximation provides a reasonable description of the IR/Raman spectra of organic crystals in the low-frequency spectral range [29,30,70,71].

The experimental IR frequencies of [CRB + MLE] (1:1) in the low-frequency spectral range are compared with the theoretical values computed at different levels of approximation (PBE-D3/6-31G(d,p), B3LYP/6-31G(d,p), B3LYP-D2/6-31G(d,p), B3LYP-D3/6-31G(d,p), and PBE-D3/PW) with fixed unit cell parameters (AtomOnly) and full unit cell relaxation (FullOpt) and the values are collected in Table 2. The periodic DFT calculations in all the approximations used produce reasonable values of vibration frequencies. The IR intensities are reproduced by all the approximations only semi-quantitatively. The use of B3LYP-D3 approximation in the periodic DFT calculations leads to the termination of the IR and Raman activity computations. For this reason, B3LYP-D2 was used to calculate the IR and Raman activities.

The frequencies of the IR-active vibrations of the parent CRB crystal in the range of 400–150 cm^−1^ are reproduced well by all the approximations (Table S4, Supplementary Materials). This is due to the absence of short (strong) or ionic H-bonds in this crystal. The IR intensities are reproduced by all the approximations only semi-quantitatively.

Periodic DFT computations of molecular crystals sometimes lead to the appearance of imaginary frequencies [6,72,73]. We encountered this problem when calculating the IR/Raman spectra of [CRB + MLE] (1:1) using the PBE-D3/PW (FullOpt) approximation (see Table S5, Supplementary Materials). Unlike calculations of non-periodic systems, there is no universal recipe for solving the problem of imaginary frequencies appearing in periodic calculations. This problem is usually solved by reducing the space symmetry of a crystal [72,74]. Other methods include (i) the use of extended basis sets [73], (ii) variation of the cell parameters [74], and (iii) increasing the atomic displacement value in numerical second derivative calculations [41]. However, in some cases, these tricks fail to result in a stable structure.

### 2.5. Raman Spectrum in the Low-Frequency Region

**Figure 3:**
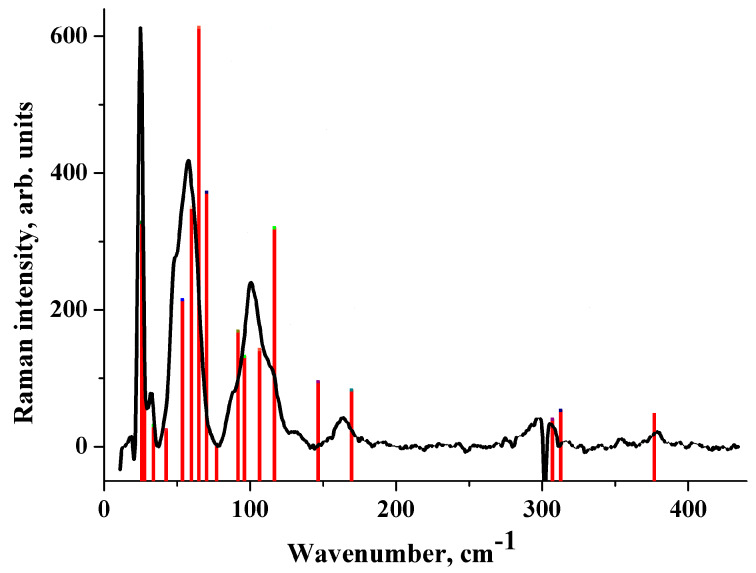
Raman spectrum of [CRB + MLE] (1:1). Experiment (black line) vs. B3LYP(AtomOnly) computations (red bars). The height of the bars is proportional to the relative Raman intensity of the corresponding transition.

The wavenumber of the lowest Raman-active vibration of [CRB + MLE] (1:1) is ~25 cm^−1^ (Figure 3). Its theoretical wavenumber is very sensitive to the level of approximation (Table S5, Supplementary Materials). The optimization of the cell parameters greatly affects this value, as well as the number of IR/Raman active vibrations below 100 cm^−1^. A significant decrease in the cell volume (about 10%) as a result of optimization leads to a blue shift in the wave number of the lowest IR/Raman active vibration by ~10 cm^−1^, in accordance with References [75,76].

The experimental Raman spectrum of [CRB + MLE] (1:1) in the low-frequency region is shown in Figure 3. The dips in the spectrum at 20.2 cm^−1^ and at 302 cm^−1^ are the artefacts of the measurements associated with the presence of dust particles on the mirrors. B3LYP with the fixed cell parameters provides a reasonable description of the Raman spectrum of [CRB + MLE] (1:1) (Figure 3). This applies to both the wave numbers and the Raman intensities. In contrast to B3LYP(AtomOnly), B3LYP-D2(FullOpt) does not provide an adequate description of the Raman spectrum (Figure S4, Supplementary Materials). PBE-D3(FullOpt) reproduces the Raman spectrum of the salt somewhat better than B3LYP-D2(FullOpt) (see Figures S4 and S5, Supplementary Materials). However, the calculated wavenumbers of the most intense bands in the region below 100 cm^−1^ are blue-shifted compared with the experiment, and the Raman intensity of the vibrations in the region of 100–400 cm^−1^ turns out to be very high. This result can be explained by a significant reduction in the cell volume of [CRB + MLE] (1:1) as a result of full optimization (see Table 2).

**Figure 4:**
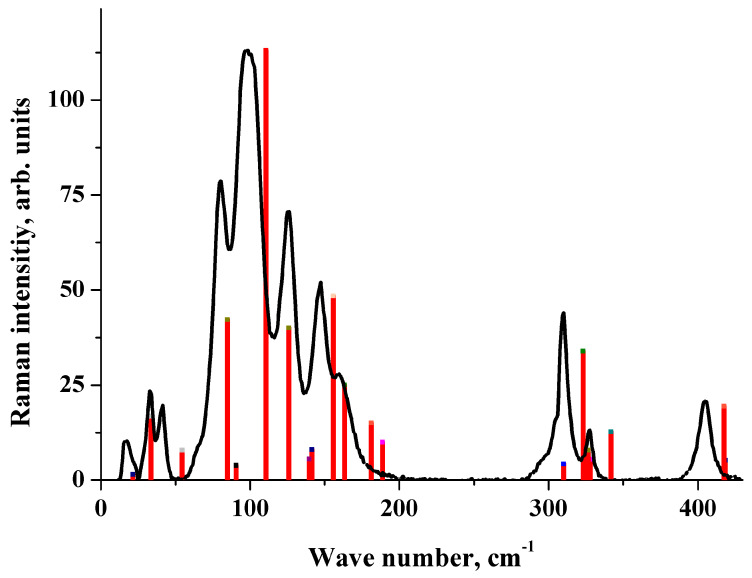
Raman spectrum of crystalline maleic acid. Experiment (black line) vs. B3LYP(AtomOnly) computations (red bars). The height of the bars is proportional to the relative Raman intensity of the corresponding transition.

In the Raman spectrum of crystalline maleic acid (Figure 4), the most intense band lies in the region of 100 cm^−1^, while the lowest Raman-active vibration is most intense in [CRB + MLE] (1:1). The B3LYP(AtomOnly) approximation reproduces these differences. This approximation provides a reasonable description of the acid Raman spectrum (Figure 4). PBE-D3(FullOpt) and B3LYP-D2(FullOpt) do not reproduce the acid Raman spectrum (Figures S6 and S7, Supplementary Materials).

**Figure 5:**
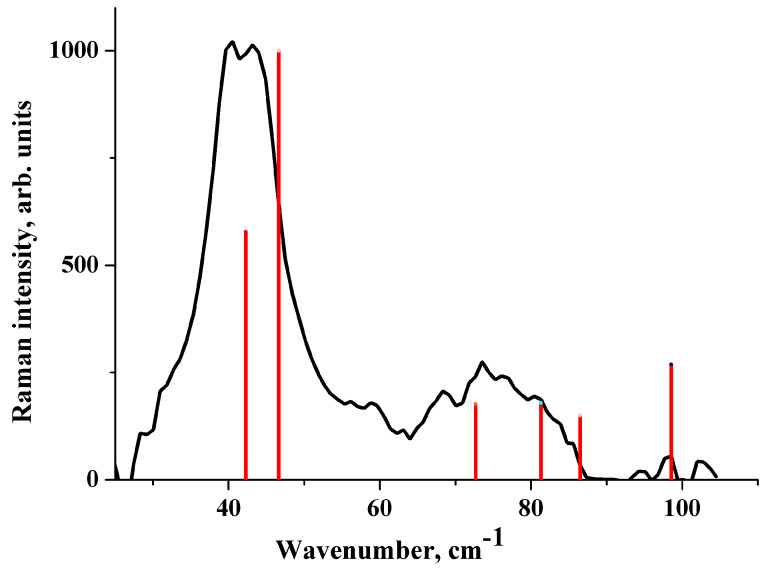
Raman spectrum of the CRB crystal in the region of 25–100 cm^−1^ (see text). Experiment (black line) vs. B3LYP(AtomOnly) computations (red bars). The height of the bars is proportional to the relative Raman intensity of the corresponding transition.

The signal from the CRB crystal contains a strong (apparently luminescent) background, and its Raman spectrum is very noisy (Figure S8, Supplementary Materials). Therefore, we focus on reproducing the spectrum below 100 cm^−1^, i.e., in the THz region. The B3LYP(AtomOnly) approximation reproduces the position of the most intense low-lying vibration and provides a reasonable description of the spectrum in the considered frequency region (Figure 5). B3LYP-D2(FullOpt) and PBE-D3(FullOpt) do not reproduce the position of the most intense low-lying vibration (Figures S9 and S10, Supplementary Materials).

**Figure 6:**
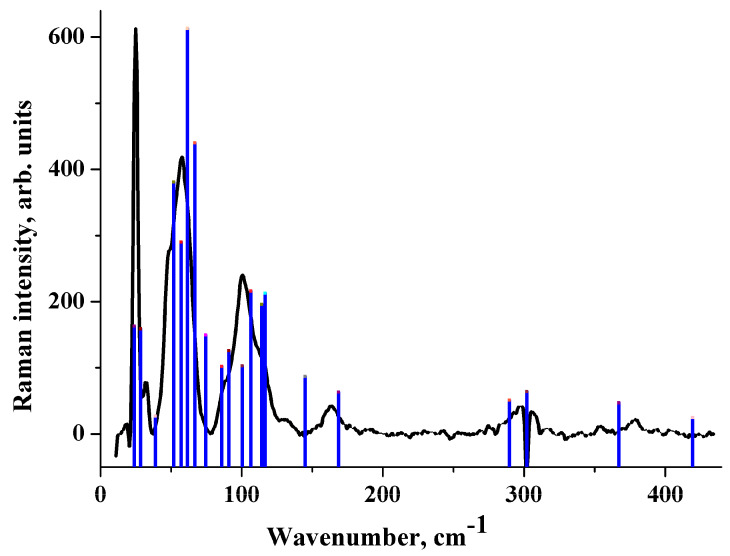
Raman spectrum of [CRB + MLE] (1:1). Experiment (black line) vs. PBE-D3(AtomOnly) computations (blue bars). The height of the bars is proportional to the relative Raman intensity of the corresponding transition.

To clarify the effect of cell parameter optimization on the Raman spectrum in the low-frequency region, calculations were performed in the PBE-D3(AtomOnly) approximation. Note that this approximation is used in periodic DFT computations with both GTO [77] and PW [78] basis sets. According to Figure 6, Figures S6 and S10 (Supplementary Materials), PBE-D3(AtomOnly) provides a reasonable description of the Raman spectrum of [CRB + MLE] (1:1) and crystals of pure CRB and MLE.

We conclude that the approximations using cell parameter optimization cannot satisfactorily describe the low-frequency Raman spectrum of the crystals with intra- and intermolecular H bonds of different strengths. This is due to the overestimation of the thermal expansion of the crystals by PBE-D3 and B3LYP-D2 (Table 2 and Table S6, Supplementary Materials). The relative changes in the cell volume for B3LYP-D2(FullOpt) are more than 10%; therefore, this approximation provides a poor description of the low-frequency Raman spectra.

The results obtained in this work show that the low-frequency Raman spectra of organic crystals with intramolecular O–H···O, intermolecular O–H···N and ^−^O···H–N^+^ bonds are reproduced in the approximations B3LYP(AtomOnly) and PBE-D3(AtomOnly). According to References [31,77], the structure and IR/Raman spectra of crystals containing organic fluorine and non-conventional C–H···F/C–H···O bonds are adequately described by PBE-D3 and modified PBE functionals with modest basis sets in terms of the AtomOnly approximation.

### 2.6. Lattice Energy Evaluation

A number of computational approaches to *E_latt_* assessment are reported in the literature. They mostly concern single-component crystals and two-component crystals without an intermolecular proton transfer (cocrystals), and they use either careful quantum chemical modeling [6,79,80,81,82] or semi-empirical schemes [23,24,83,84,85,86]. Molecular salts present new challenges for those developing theoretical approaches describing the lattice energy *E_latt_*. These crystals consist of closed-shell organic ions interacting through ionic H-bonds, which may be partially covalent [21,87]. This is one of the reasons for high *E_latt_* values in two-component organic crystals, e.g., 246 kJ·mol^−1^ (Reference [88]), 259–286 kJ·mol^−1^ (Reference [89]), 272 kJ·mol^−1^ (Reference [18]), 253–295 kJ·mol^−1^ (Reference [90]), 299 kJ·mol^−1^ (Reference [91]), etc. It should be pointed out that the *E_latt_* values for one-component crystals included in the benchmark sets vary from 25 kJ·mol^−1^ for CO_2_ to 163 kJ·mol^−1^ for cytosine [5]. The intermolecular proton transfer occurs only in the condensed phase [92], which means that there are closed-shell ions in a molecular crystal (BH^+^ cation and A^−^ anion), and neutral organic molecules B and HA in the gas phase. Gas-phase energies of these species (*E_mol_*) are different for neutral molecules and closed-shell ions. This leads to some ambiguity in the choice of gas-phase structures during the *E_latt_* calculation (see Equations (1) and (2) in Section 3.6). In our case, these structures can be either a CRBH^+^ cation and a maleate ion, or molecules of carbendazim and maleic acid (see the Supplementary Materials for the calculation details).

GTO basis sets require evaluation of the correction of the basic set superposition error (BSSE) (Equation (2)). The Crystal17 evaluation scheme for this correction involves only neutral molecules. It was assumed that BSSEs for the neutral molecules are equal to the values of BSSE for the ionic species in [CRB + MLE] (1:1). The *E_latt_* value for the neutral molecules in the gas phase was found to be around 250 kJ·mol^−1^ (Table 3), which is comparable to the known *E_latt_* values for multicomponent pharmaceutical crystals estimated using other schemes [45,93,94]. The *E_latt_* values obtained by PBE-D3 with and without variation of cell parameters agree well with each other.

In addition, they corresponded to *E_latt_* computed by PBE-D3/PW. The lattice energies obtained with the CRBH^+^ cation and maleate ion in the gas phase were found to be above 600 kJ·mol^−1^ (Table 3). Moreover, this value depended on the DFT functional and the applied BSSE correction. Such large *E_latt_* values for multicomponent crystals of organic salts are known in the literature [95,96,97,98,99]. It should be noted that there is a special class of one-component crystals consisting of zwitterionic molecules, which are also characterized by large *E_latt_* values [33]. We have to admit that the scheme we used for *E_latt_* evaluation of crystals of organic salts requires further development. The raised problem of accounting for BSSE for organic salts and uniform description of the species in crystals and gas phase originates from two independent sources: (1) an assumption of additivity of BSSE corrections for multicomponent crystals; (2) limited ability of the existing approaches to treat crystals of organic salts, e.g., BH^+^ and A^−^ in a crystal and B and HA in the gas phase.

In summary, the optimization of cell parameters does not lead to a noticeable change in the *E_latt_* value despite the significant variation of the cell volume. The use of the GTO and PW basis sets in the PBE-D3 approximation leads to close *E_latt_* values.

## 3. Materials and Methods

### 3.1. Compounds and Solvents

Carbendazim (C_9_H_9_N_3_O_2_, 98%) was purchased from Acros Organics (Geel, Belgium), and maleic acid (C_4_H_4_O_4_, 98%) was purchased from Merck (Darmstadt, Germany). The solvents were purchased from different suppliers and were used as received without further purification.

### 3.2. Cocrystal Preparation

The grinding experiments were performed using a Fritsch planetary micro mill (Fritsch, Idar-Oberstein, Germany), model Pulverisette 7, in 12-mL agate grinding jars with ten 5-mm agate balls at a rate of 500 rpm for 40 min. In a typical experiment, 80–100 mg of the carbendazim/maleic acid mixture in the 1:1 stoichiometric ratio was placed into a grinding jar, and 40–50 μL of methanol was added with a micropipette. In the other method, 200 mg of the 1:1 mixture of carbendazim and maleic acid was suspended in 2 mL of methanol and left to be stirred overnight on a magnetic stirrer at room temperature. The precipitate was filtered from the solution and dried at room temperature.

The diffraction-quality single crystals of [CRB + MLE] (1:1) were obtained by dissolving 90 mg of the stoichiometric 1:1 mixture of the components in 5 mL of methanol at 40 °C. After complete dissolution, the solution was gently cooled to room temperature; then, it was covered with Parafilm with a few small holes pierced in it and left for the solvent to evaporate. After a week, small colorless crystals appeared in the solution.

### 3.3. Thermal Analysis

The thermal analysis was carried out using a differential scanning calorimeter (DSC) with a refrigerated cooling system (Perkin Elmer DSC 4000, Perkin Elmer Inc., Waltham, MS, USA). The sample was heated in a standard sealed aluminum pan (40 μL volume) at a rate of 10 °C·min^−1^ in a nitrogen atmosphere. The unit was calibrated with indium and zinc standards. The accuracy of the weighing procedure was ±0.01 mg. The results of the DSC analysis for [CRB + MLE] (1:1) and pure components are presented in Figure S11 (Supplementary Materials).

### 3.4. Single-Crystal and Powder X-ray Diffraction (XRD) Experiments

Single-crystal XRD data were collected on a SMART APEX II diffractometer (Bruker AXS, Karlsruhe, Germany) using graphite-monochromated Mo*K*_α_ radiation (*λ* = 0.71073 Å) at 120 and 296 K. Absorption corrections based on the measurements of equivalent reflections were applied [100]. The structures were solved by direct methods and refined by full matrix least-squares on *F*^2^ with anisotropic thermal parameters for all the non-hydrogen atoms [101]. All the hydrogen atoms were found from a difference Fourier map and refined isotropically. The crystallographic data for [CRB + MLE] (1:1) were deposited by the Cambridge Crystallographic Data Center (Cambridge, UK) as supplementary publications under the CCDC numbers 1,994,877 and 1,994,878 for 120 and 296 K, respectively. This information can be obtained free of charge from the Cambridge Crystallographic Data Center via www.ccdc.cam.ac.uk/data_request/cif.

The X-ray powder diffraction (PXRD) data of the bulk materials were recorded under ambient conditions in Bragg–Brentano geometry on a Bruker D2 Phaser diffractometer equipped with a second-generation LynxEye detector (Bruker AXS, Karlsruhe, Germany) with CuKα radiation (*λ* = 1.5406 Å). PXRD patterns of salt and parent solids are given in Figure S12 (Supplementary Materials).

### 3.5. IR and Raman Spectroscopy

The Fourier-transform infrared (FT-IR) spectra of the compounds were recorded in the spectral range of 400–150 cm^−1^ from CsBr pellets on a Bruker Vertex 80 V device (Bruker Optik, Ettlingen, Germany) equipped with a Mylar multilayer beamsplitter. The high-quality spectra were obtained and analyzed using the OPUS 6.5.83 software (Bruker Optik, Ettlingen, Germany).

The Raman measurements in the spectral range of 10–440 cm^−1^ were performed using a Raman microscope (inVia, Renishaw plc, Spectroscopy Product Division, Old Town Wotton-Under-Edge, Gloucestershire, GL12 7DW, UK) with a 50× objective lens (Leica DM 2500 M, NA = 0.75, Leica Mikrosysteme Vertrieb GmbH, Mikroskopie und Histologie Ernst-Leitz-Strasse 17-37Wetzlar, 35578 Germany). The measurements were made with a NExT monochromator (Renishaw plc, Spectroscopy Product Division, Old Town Wotton-Under-Edge, Gloucestershire, GL12 7DW, UK). The excitation wavelength was 633 nm, as provided by an He–Ne laser (RL633, Renishaw plc, Spectroscopy Product Division, Old Town Wotton-Under-Edge, Gloucestershire, GL12 7DW, UK) with the maximum power of 17 mW. The acquisition time and number of accumulations were adjusted to maximize the signal-to-noise ratio with the minimal sample degradation. All the spectra for the powder samples were measured at several points and then averaged to reduce the anisotropy effect on the Raman spectra and to increase the single-to-noise ratio. The background from the Raman spectra was subtracted by the cubic spline interpolation method. All the spectra were divided by the number of accumulations and acquisition time. The dips in the spectra at wavenumbers of 20.2 cm^−1^ and 302 cm^−1^ are the artefacts of the measurements associated with the presence of dust particles on the NExT monochromator mirrors.

### 3.6. Periodic (Solid-State) DFT Computations

In the CRYSTAL17 calculations [102], we employed the B3LYP [103,104] and PBE [105] functionals with the all-electron Gaussian-type localized orbital basis sets 6-31G(d,p). The London dispersion interactions were taken into account by introducing the D3 correction with Becke–Jones damping (B3LYP-D3 and PBE-D3) and the D2 correction (B3LYP-D2) developed by Grimme et al. [106,107]. In the QuantumExpresso calculations [108,109], we employed PBE with a plane-wave basis set. PAW pseudopotentials with a cut-off energy of 100 Ry were used [110]. The London dispersion interactions were taken into account by introducing the D3 correction with Becke–Jones damping (PBE-D3). In one series of calculations, the space groups and the unit cell parameters of the crystals obtained from the X-ray diffraction experiment were fixed, and the structural relaxations were limited to the positional parameters of the atoms (AtomOnly). In the other series, the optimization was also performed by the cell parameters without cell volume restrictions (FullOpt). The symmetry of crystals was kept during all computations.

The crystal lattice energy *E_latt_* of the *n*-component crystal was estimated from periodic DFT as the difference between the sum of total electronic energies of relaxed isolated species *E_mol_* and the total energy of the crystal *E_cry_* calculated per asymmetric unit [111]

(1) $$E_{latt}=\sum_{i=1}^{n}E_{mol,i}-\frac{E_{cry}}{Z}$$

Equation (1) was used in PBE/PW calculations. In the case of GTO basis sets, the basis set superposition error (BSSE) [112] was taken into account.

(2) $$E_{latt}=\sum_{i=1}^{n}\left(E_{mol,i}+BSSE_{i}\right)-\frac{E_{cry}}{Z}$$

Further details of the calculations are given in Section S1 of the Supplementary Materials.

## 4. Conclusions

In this work, we investigated the influence of cell parameter optimization on the *E_latt_* value, as well as the structural and spectroscopic properties of the new two-component carbendazim maleate crystal. The sign and absolute value of the relative change in the cell volume of the crystal depends on the functional and type of Grimme correction. Some properties of the considered crystal (metric parameters of short/ionic H-bonds, low-frequency Raman spectra) strongly depend on the changes in the cell volume, while other properties (lattice energy *E_latt_*, infrared spectra in the 400–150 cm^−1^ frequency region) are weakly related to these variations.

Optimization of the cell parameters of [CRB + MLE] (1:1) and crystals made up of its constituents greatly affects the wavenumber of the lowest Raman-active vibration, the number of Raman active vibrations below 100 cm^−1^, and the relative intensity of these vibrations. B3LYP and PBE-D3 with fixed cell parameters provide a reasonable description of the low-frequency Raman spectra of the considered molecular crystals. This applies both to the wave numbers and Raman intensities. B3LYP and PBE-D3 with modest basis sets and fixed cell parameters can be recommended for evaluation of the structure, H-bond pattern, and infrared/Raman spectra of multicomponent pharmaceutical crystals.

The applicability of different DFT approximations to the *E_latt_* calculation of the pharmaceutical salt of carbendazim and maleic acid was examined. It is shown that the existing methods for the calculation of *E_latt_* require further developments for solving the problem of accounting for BSSE for organic salts and uniform description of the species in crystals (closed-shell organic ions) and gas phase (neutral organic molecules). It is shown that optimization of the cell parameters does not lead to a noticeable change in the *E_latt_* value despite the significant variation of the cell volume. The use of the GTO and PW basis sets in the PBE-D3 approximation leads to close *E_latt_* values.

The PBE-D3 method with modest basis sets and fixed cell parameters provides a reasonable trade-off between the accuracy and the computational cost in evaluation of a number of relevant properties of multicomponent pharmaceutical crystals.

## Figures and Tables

**Table 1:** Distances between the heavy atoms involved in the formation of conventional hydrogen bonds in [CRB + MLE] (1:1). Experiment (Exp.) vs. theoretical values. Computations at different levels of approximation with fixed unit cell parameters (AtomOnly) and full unit cell relaxation (FullOpt). The FullOpt values are given in parentheses. The units are Å. The relative change in the volume of the crystallographic cell ∆*V* is given in the last line.

Fragment ^(a)^	Exp.	Computations				
		PBE-D3/6-31G(d,p)	B3LYP/6-31G(d,p)	B3LYP-D2/6-31G(d,p)	B3LYP-D3/6-31G(d,p)	PBE-D3/PW ^(b)^
O24–H24···O21 (intra-)	2.442	2.462 (2.481)	2.446 (2.399)	2.468 (2.433)	2.457 (2.443)	2.453 (2.456)
N1–H1···O21	2.685	2.662 (2.700)	2.665 (2.622)	2.700 (2.640)	2.680 (2.641)	2.675 (2.670)
N3–H3···O22	2.761	2.735 (2.798)	2.768 (2.718)	2.762 (2.746)	2.757 (2.745)	2.741 (2.744)
N2–H2···O23	2.756	2.701 (2.665)	2.714 (2.684)	2.750 (2.681)	2.701 (2.677)	
∆V = (V_exp_ − V_theor_)/V_exp_ (%)	3.1	6.8	−9.2	13.3	9.4	<−0.1

^(a)^ Atomic numbering is given in Figure 2; ^(b)^ PW stands for the plane-wave basis set with a cut-off energy of 100 Ry and PAW pseudopotentials.

**Table 2:** Frequencies and infrared (IR) intensities of the low-frequency vibrations of [CRB + MLE] (1:1). Experiment vs. computations at different levels of approximation. The values obtained using the FullOpt option are shown in italics. The units are cm^−1^ (wave numbers) and kM/mol (intensities).

Exp. ^(a)^	Normal Mode Symmetry, Assignment ^(b)^	Computations ^(c)^			
		PBE-D3/6-31G(d,p)	B3LYP/6-31G(d,p)	B3LYP-D2/6-31G(d,p)	PBE-D3/PW ^(d)^
180–167 vs, broad	B_u_, ν(N3···O22) + ν(N1···O21)	179 (453) 186 (336) ^(e)^	180 (353) 171 (390)	150 (66) ^(e)^	177
215 s	B_u_, CH_3_ twist	219 (62) 228 (80)	222 (63) 219 (51)	202 (313)	
266 s	B_u_, ν(O24···O21)	257 (172) 260 (194)	264 (165) 263 (153)	278 (152)	258
302 s	B_u_, CNC(=O) bending	299 (58) 302 (43)	303 (68) 302 (65)	312 (95)	
330–346 vs, broad	B_u_, ν(N1···O21)	362 (132) 365 (46)	358 (223) 334 (122)	368 (156)	349
380 s	B_u_, ν(N3···O22)	368 (160) 376 (206)	374 (76) 351 (183)	383 (88) ^(e)^	364

^(a)^ the abbreviations used for relative intensities are vs, very strong; s, strong; ^(b)^ Atomic numbering is given in Figure 2; ^(c)^ the IR intensities are given in parenthesis; ^(d)^ PW stands for the plane-wave basis set with a cut-off energy of 100 Ry and PAW pseudopotentials; ^(e)^ in the calculations, this is a doublet of bands with almost identical wave numbers and IR intensities.

**Table 3:** Crystal lattice energy of [CRB + MLE] (1:1) derived from the periodic DFT computations with plane wave and Gaussian-type orbitals. ^a^ The units are kJ·mol^−1^.

	B3LYP-D3/6-31G(d,p) (AtomOnly)	PBE-D3/6-31G(d,p) (AtomOnly)	PBE-D3/6-31G(d,p) (FullOpt)	PBE-D3/PW (AtomOnly)
Neutral molecules in the gas phase	277.8	266.8	258.4	278.3
Charged ions in the gas phase ^a^	817.2	655.6	647.2	625.0

^a^ See the Supplementary Materials.

## Supplementary Materials

The following are available online: Section S1. Computational details; Table S1. Crystallographic data for [CRB + MLE] (1:1); Table S2. Experimental metric parameters and interaction energies of conventional intermolecular hydrogen bonds in [CRB + MLE] (1:1); Table S3. Experimental vs. theoretical distances between heavy atoms involved in the formation of nonconventional hydrogen bonds and C-H-O bond angles in [CRB + MLE] (1:1); Table S4. Tentative assignment of the bands in the IR spectrum of the pure CRB crystal below 400 cm^−1^; Table S5. Calculated values of low frequency IR or Raman active phonons in [CRB + MLE] (1:1) and the volume of the crystallographic cell; Table S6. Volume of the crystallographic cell of the CRB and MLE crystals after optimization; Figure S1. Part of the crystal structure with notable non-conventional hydrogen bonds; Figure S2. Experimental and theoretical IR spectra in the low-frequency range; Figure S3. Experimental and theoretical IR spectra of [CRB + MLE] (1:1) in high-frequency and mid-frequency range; Figure S4. Experimental vs. theoretical [B3LYP-D2(FullOpt)] Raman spectrum of [CRB + MLE] (1:1); Figure S5. Experimental vs. theoretical [PBE-D3(FullOpt)] Raman spectrum of [CRB + MLE] (1:1); Figure S6. Experimental vs. theoretical [PBE-D3(AtomOnly) and PBE-D3(FullOpt)] Raman spectrum of MLE; Figure S7. Experimental vs. theoretical [B3LYP-D2(FullOpt)] Raman spectrum of MLE; Figure S8. Experimental Raman spectrum of CRB; Figure S9. Experimental vs. theoretical [B3LYP-D2(FullOpt)] Raman spectrum of CRB in the THz region; Figure S10. Experimental vs. theoretical [PBE-D3(AtomOnly) and PBE-D3(FullOpt)] Raman spectrum of CRB in the THz region; Figure S11. Results of DSC analysis of [CRB + MLE] (1:1) and its pure components; Figure S12. Powder X-Ray diffraction patterns for pure CRB, MLE, and [CRB + MLE] (1:1) (experimental and theoretical).
