# Supplementary material for: Combined X-ray Crystallographic, IR/Raman Spectroscopic, and Periodic DFT Investigations of New Multicomponent Crystalline Forms of Anthelmintic Drugs: A Case Study of Carbendazim Maleate
Source: Molecules. 2020 May 21;25(10):2386. doi: 10.3390/molecules25102386 (PMC7287603; doi:10.3390/molecules25102386)
Supplement: Supplementary file 1 [file molecules-25-02386-s001.pdf]

Electronic supporting information

Combined X-Ray crystallographic, IR/Raman spectroscopic and periodic DFT investigations of new multicomponent crystalline forms of anthelmintic drugs: a case study of carbendazim maleate.

Alexander P. Voronin [a], Artem O. Surov [a], Andrei V. Churakov [b], O. D. Parashchuk [c], Alexey A. Rykounov [d], Mikhail V. Vener*[e]

[a] *G.A. Krestov Institute of Solution Chemistry of RAS, Ivanovo, Russia*

[b] *N.S. Kurnakov Institute of General and Inorganic Chemistry of RAS, Moscow, Russia*

[c] *Faculty of Physics, Lomonosov Moscow State University, Moscow, Russia*

[d] *FSUE "RFNC-VNIITF named after Academ. E.I. Zababakhin", Snezhinsk, Russia*

[e] *D. Mendeleev University of Chemical Technology, Moscow, Russia*

**Corresponding author*: Mikhail V. Vener, e-mail: mikhail.vener@gmail.com

Table of Contents:

Section	Page
S1. Computational details	S2-S4
Table S1	S4
Table S2	S4
Table S3	S5
Table S4	S6
Table S5	S7
Table S6	S7
Figure S1	S8
Figure S2	S8
Figure S3	S9
Figure S4	S10
Figure S5	S10
Figure S6	S11
Figure S7	S11
Figure S8	S12
Figure S9	S12
Figure S10	S13
Figure S11	S13
Figure S12	S14
References	S15

S1. Computational details

S1.1 Periodic (solid-state) DFT calculations and lattice energy calculation

Periodic DFT computations with all-electron Gaussian-type orbitals (GTO) were performed using Crystal17 [1]. The 6-31G** basis set was used. The tolerance on energy controlling the self-consistent field convergence for geometry optimizations and frequency computations was set to 10^{-10} and to 10^{-11} Hartree, respectively. The number of points in the numerical first-derivative calculation of the analytic nuclear gradients equals 2. The shrinking factor reflecting the density of the k-points grid in the reciprocal space was set to 4. *K*-space sampling was limited to the Γ point. Raman intensities were calculated using the "RAMANEXP" keyword. Temperature was 298 K, the frequency of the incoming laser was 633 nM.

Experimental crystal structures of [CRB+MLE] (this work), CRB (refcode SEDZUW01) and MLE (MALIAC12) were used as input for all geometry optimization computations, with hydrogen atom positions normalized to standard neutron diffraction values. All the optimized structures were found to correspond to the minimum point on the potential energy surface.

DFT calculations in plane-wave basis set were done with Quantum ESPRESSO v. 6.3 program package [2,3]. For all atoms pseudopotentials from pslibrary-1.0.0 (http://quantum-espresso.org, Andrea Dal Corso, 2013) were used. Cut-off energy was set to 100 and 400 Ry for wavefunction and electron density correspondingly.

The crystal lattice energy E_{latt} of the n-component crystal was estimated from periodic DFT as the difference between the sum of total electronic energies of isolated species in their relaxed conformations E_{mol} and the total energy of the crystal E_{cry} calculated per asymmetric unit:

$$E_{latt} = \sum_{i=1}^{n} E_{mol,i} - \frac{E_{cry}}{Z} \quad \text{(s1)}$$

Isolated species can be both neutral molecules and ions. Eq. (s1) was used in PBE/PW calculations.

For isolated molecules and ions in periodic DFT calculations with plane-wave basis set was used cubic box with edge length 60 a.u. The cut-off energies were the same as in calculations of crystal.

Lattice energy calculation using GTO require taking into account the basis set superposition error (BSSE), which requires modification of eq. (s1):

$$E_{latt} = \sum_{i=1}^{n} (E_{mol,i} + BSSE_i) - \frac{E_{cry}}{Z} \quad \text{(s2)}$$

Computations of E_{mol} were performed in CRYSTAL17 using the keyword MOLECULE. BSSE was computed using the Boys–Bernardi counterpoise correction scheme with the

MOLEBSSE option [4]. The standard molecular extraction procedure built in CRYSTAL17 does not treat the charged species correctly. This leads to the situation where ions exist instead of neutral molecules. For single point SCF calculations and geometry relaxation, no problems occurred; however, BSSE calculations of ions failed with following error message:
ERROR **** GHOSTD **** MOLECULE - SET UHF AND SPINLOCK - ODD NUMBER OF ELECTRONS

We followed the recommendation of the program and added UHF and SPINLOCK 2 to the input file for MOLEBSSE calculation. This resulted in normal program termination. In order to obtain comparable SCF energies for neutral molecules in gas phase, SCF computations without counterpoise correction were also performed using UHF/SPINLOCK 2 keywords, and BSSE correction was calculated as the difference between the total electronic energies of MOLEBSSE and SCF computations.

The computations using eq. (s2) with neutral molecules in the gas phase were not 'straightforward' as well. Since we could not transform an ion to the corresponding neutral molecule by program means, the molecular structures of pure components (extracted from crystalline CRB/ refcode SEDZUW01, and MLE/ refcode MALIAC12) were used as initial points for E_{mol} calculation. We had to consider BSSE energy for molecular species equal to that for ionic species calculated in UHF approximation.

S1.2. Non-covalent interaction energies

In order to quantify the energies of particular non-covalent interactions in crystal, Bader analysis of crystalline electron density was performed in the TOPOND software [5] currently built into CRYSTAL suit. The search for (3;-1) critical points was conducted between the pairs of atoms within the 5Å radius, and the interactions with electron density ρ_b in the (3;-1) point higher than 0.003 a.u. were taken for consideration.

The energy of the particular noncovalent interaction, E_{int}, was evaluated from the local kinetic energy density in the (3;-1) critical point (G_b) according to following equation [6]:

$$E_{int}\ (\text{kJ mol}^{-1}) = 1147\ G_b\ (\text{a.u.}) \qquad (s3)$$

Eq. (2) yields reasonable E_{int} values for molecular crystals with different types of intermolecular interactions: H-bonds, C−H···O, Hal···Hal contacts, etc. [7-9]. The additive scheme based on equation (2) provides lattice energies that are close to experimental sublimation enthalpies for single- and multicomponent molecular crystals, as shown in Refs. [10-12]

Energies of conventional intermolecular H-bonds were also estimated by several correlation equations based either on metric or spectroscopic parameters. For instance, Rozenberg equation [13] (s3) allows estimating the hydrogen bonding energy E_{HB} (kJ mol^{-1}) in liquids, solids and solutions using only the distance between hydrogen and acceptor atoms R_{HA} expressed in nm:

$$E_{HB} = 0.134 \cdot (R_{HA})^{-3.05} \quad \text{(s4)}$$

Equation (s4) applied in DREIDING/A force field proposed by Mayo et al.[14] is based on CHARMM-like hydrogen-bonding potential:

$$E_{HB} = D_{hb}\left[5(R_{hb}/R_{DA})^{12} - 6(R_{hb}/R_{DA})^{10}\right] \cdot \cos^4(\theta_{DHA}) \quad \text{(s5)}$$

Here D_{hb} = 39.75 kJ mol^{-1}, R_{hb} = 2.75 Å, R_{DA} is the distance between heavy atoms involved in hydrogen bonding in angstroms.

Table S1. Crystallographic data for [CRB+MLE] (1:1) recorded at 120 K and 296 K.

Compound reference	[**CRB+MLE**] (1:1)	
Chemical formula	$C_9H_{10}N_3O_2 \cdot C_4H_3O_4$	$C_9H_{10}N_3O_2 \cdot C_4H_3O_4$
*F*w	307.26	307.26
Crystal system	Monoclinic	Monoclinic
a, Å	9.0642(2)	9.1087(4)
b, Å	7.3751(1)	7.1537(3)
c, Å	22.0127(4)	21.9286(9)
β, °	100.9767(7)	101.451(1)
Unit cell volume, Å^3	1444.61(5)	1400.45(10)
Temperature, K	**296(2)**	**120(2)**
Space group	$P2_1/c$	$P2_1/c$
No. of formula units per unit cell, *Z*	4	4
Radiation wavelength, Å	0.71073	0.71073
$R_1[I>2\sigma(I)]$	0.0385	0.0446
wR_2	0.1026	0.1001
CCDC number	1994878	1994877

Table S2. Experimental metric parameters and interaction energies of conventional intermolecular hydrogen bonds in [CRB+MLE] (1:1) calculated using different approaches.

Interaction	D (N···O), Å	∠(N-H-O), °	E_{int}, kJ mol^{-1}		
			Eq. (s3)	Eq. (s4)	Eq. (s5)
$N1^{+}$-H1···$O21^{-}$	2.6848(13)	176.1(16)	44.9	25.6	37.8
N3-H3···O22	2.7607(15)	173.8(15)	35.6	20.3	38.8
N2-H2···O23	2.7560(14)	143.5(14)	26.9	17.9	16.6

Table S3. Distances between heavy atoms involved in the formation of nonconventional hydrogen bonds and C-H-O bond angles in crystalline [CRB+MLE] (1:1). Experiment vs. theoretical values computed using different levels of approximation with fixed cell parameters (AtomOnly). The units are Å and degrees. (Values that differ from the experiment by no more than 0.1 Å and 1 °are indicated in bold.)

Fragment [a]	Exp.		PBE-D3/6-31G**		B3LYP/6-31G**		B3LYP-D2/6-31G**		B3LYP-D3/6-31G**		PBE-D3/PW [b]	
C6-H6 ··O24	**3.381**	**173.8**	3.256	175.6	3.264	172.1	3.299	**173.2**	3.276	175.1	3.303	174.7
C12-H121 ··O22	**3.279**	**132.5**	**3.271**	135.8	3.254	132.2	3.248	128.9	3.263	**132.4**	3.249	131.4
C4-H4 ··O23	**3.272**	**121.9**	3.255	120.3	3.249	**121.0**	3.237	**122.2**	3.243	**121.5**	3.287	119.9
C23-H23 ··O2	**3.510**	**151.1**	3.469	149.9	3.475	**150.7**	3.532	**150.5**	**3.517**	**150.5**	3.479	149.9

[a] Oxygen atom involved in the formation of the intra- and intermolecular H-bonds is denoted as O_b;
[b] PW stands for plane-wave basis set with cut-off energy 100 Ry. PAW pseudopotentials [15] were taken from pslibrary-1.0.0 (http://quantum-espresso.org, Andrea Dal Corso, 2013);

Table **S4**. Tentative assignment of the bands in the IR spectrum of the pure CRB crystal below 400 cm^{-1}. The units are cm^{-1} (frequencies) and kM/mol (intensities).

Exp. [c)]	Tentative assignment	Periodic computations [b)]			
		PBE-D3 AtomOnly	B3LYP AtomOnly	B3LYP-D2 FullOpt	PBE-D3/PW AtomOnly
220 m	CH_3 twist	211 (17) [g)]	217 (15) [g)]	229 (18)	218.2
273 s	O-CH_3 bending	288 (34)	275 (6)	248 (54)	258.3
295-320 vs, broad	C-N(H)-C bending	308 (55)	315(46)	318 (41)	297.1
355 m	non-planar cycle vibrations	360 (5)	373 (5)	383 (2.5)	363.7
394-404 vs, broad	C-O-CH_3 bending	393 (106)[g)]	401 (104) [g)]	-	419.0

[a)] Oxygen atom involved in the formation of the intra- and intermolecular H-bonds is denoted as O_b;
[b)] all-electron Gaussian-type orbital calculations were performed with the 6-31G** basis set;
[c)] abbreviations used for relative intensities: vs, very strong; s, strong; m, medium;
[d)] non-periodic computations of the CRB dimer in water (PCM);
[e)] IR intensities are given in parenthesis;
[f)] PW stands for plane-wave basis set with cut-off energy 100 Ry and PAW pseudopotentials;
[g)] in the calculations, this is a doublet of bands with almost identical wave numbers and IR intensities

Table **S5**. Calculated values of low frequency infrared (IR) or Raman (R) active phonons in [CRB+MLE] (1:1) and the volume of the crystallographic cell.

	PBE-D3/6-31G**		B3LYP/6-31G**		B3LYP-D2/6-31G**		B3LYP-D3/6-31G**		PBE-D3/PW	
	AtomOnly	FullOpt	AtomOnly	FullOpt	AtomOnly	FullOpt	AtomOnly	FullOpt	AtomOnly	FullOpt
Volume, Å^3	1444.6	1348.8	1444.6	1580.4	1444.6	1254.2	1444.6	1310.8	1444.6	1447.0
The lowest vibration, cm^{-1}	21.5 (IR)<br>24.0 (R)	25.0 (IR)<br>27.7 (R)	22.5 (IR)<br>26.0 (R)	13.3 (IR)<br>23.5(R)	18 (IR)<br>17 (R)	29.1 (IR)<br>29.7 (R)	19.1 (IR)<br>23.9 (R)	25 (IR)<br>28.6 (R)	23.4 (IR)<br>10.1 (R)	21.0 (IR)<br>-7.4 (R)
No. of IR active bands below 100 cm^{-1}	18	15	16	21	19	11	18	12	16	
No. of Raman active bands below 100 cm^{-1}	20	18	20	24	21	14	20	15	18	

Table S6. Volume of the crystallographic cell of the CRB and MLE crystals obtained using different approximations with the GTO basis set. Relative change in the volume of the crystallographic cell $\Delta V^{a)}$ is given in parenthesis. The units are Å.

	MLE[b]	CRB[c]
Experiment	469.3	875.4
PBE-D3(FullOpt)	438.9 (6.5)	842.1 (3.8)
B3LYP-D2(FullOpt)	411.4 (12.3)	761.8 (13.0)

[a)] $\Delta V = (V_{exp} - V_{theor})/ V_{exp}$ (%);
[b)] The structure was recorded at 180 K;
[c)] The structure was recorded at 295 K.

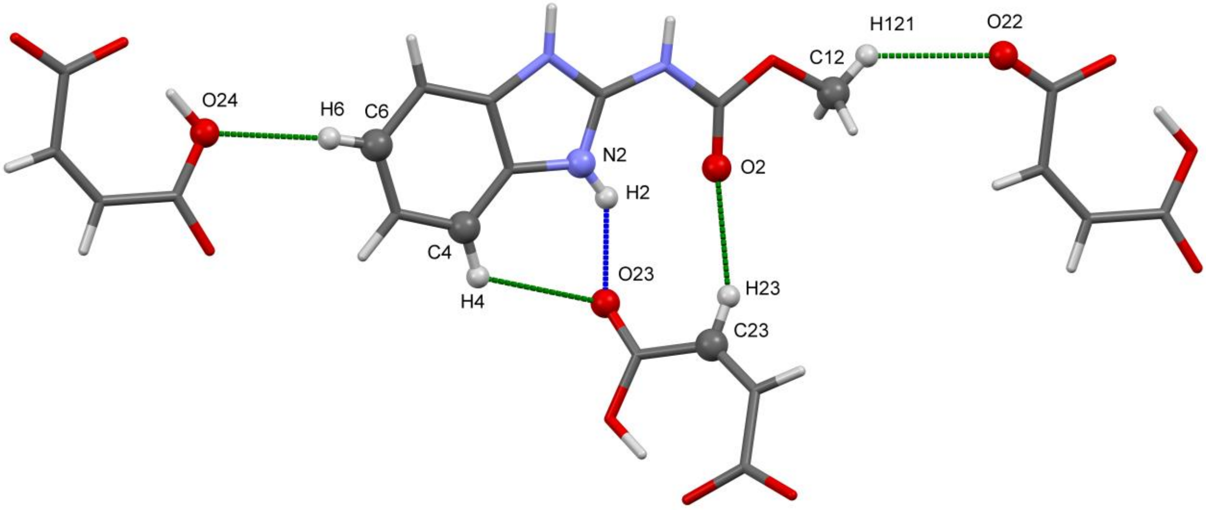

Figure S1. Part of the crystal structure with notable non-conventional H-bonds.

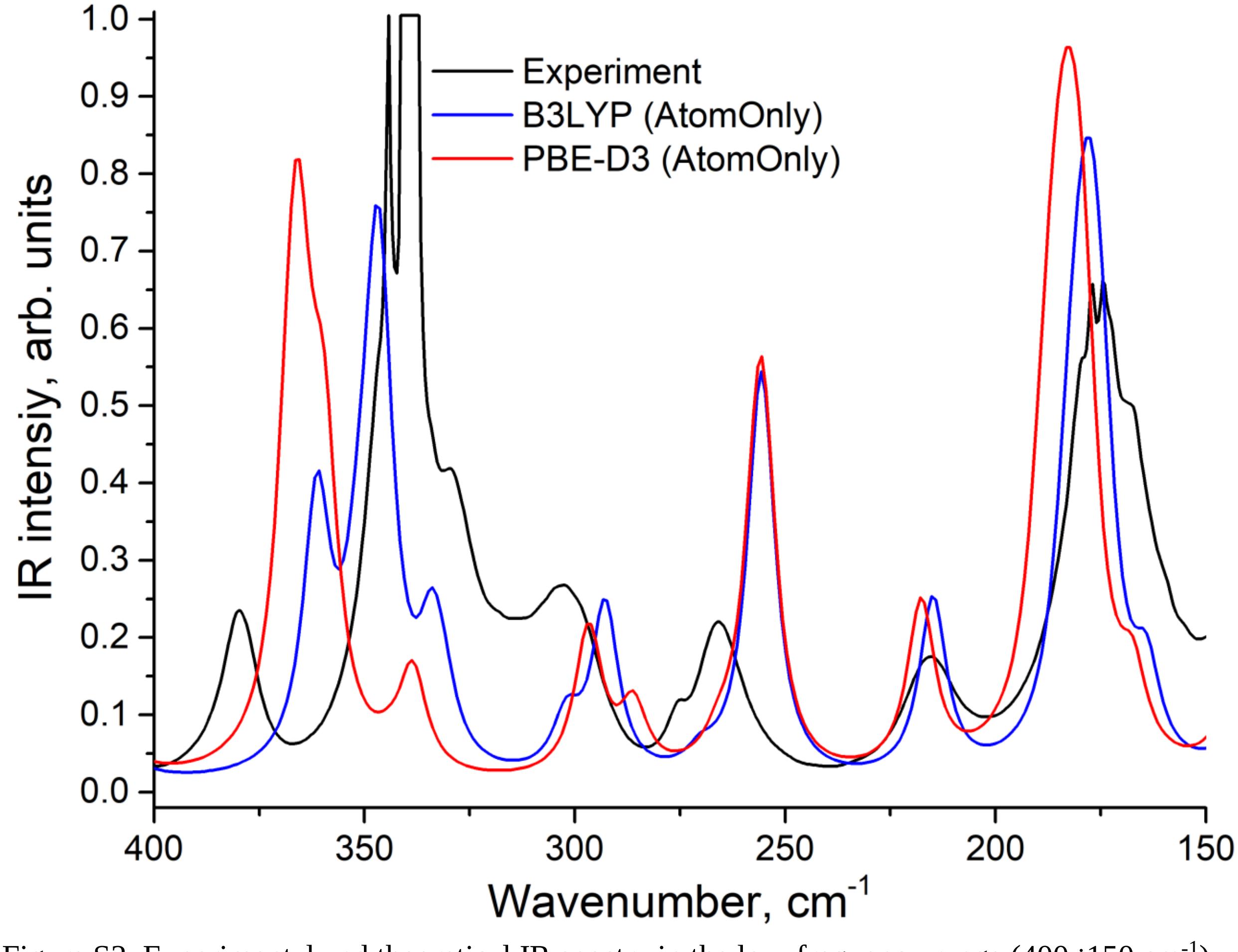

Figure S2. Experimental and theoretical IR spectra in the low-frequency range (400 ÷150 cm^{-1}).

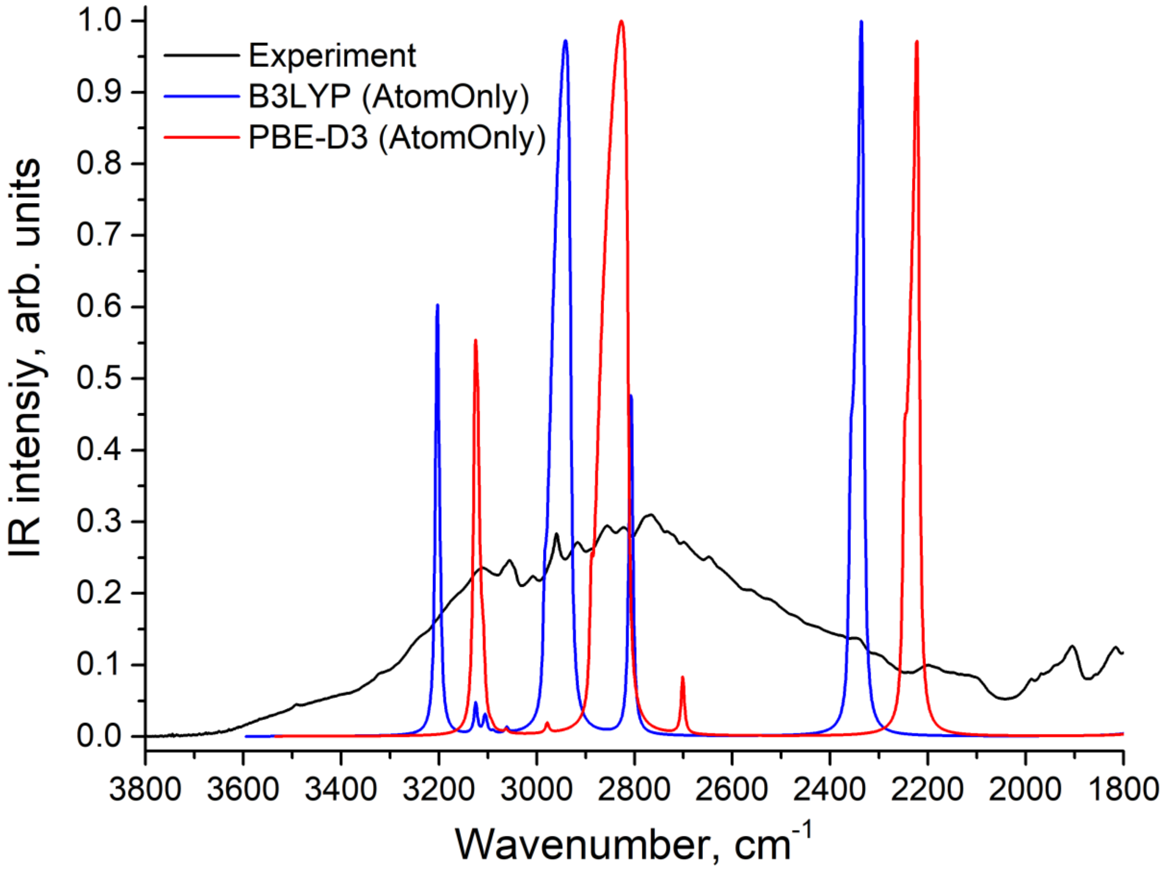

(a)

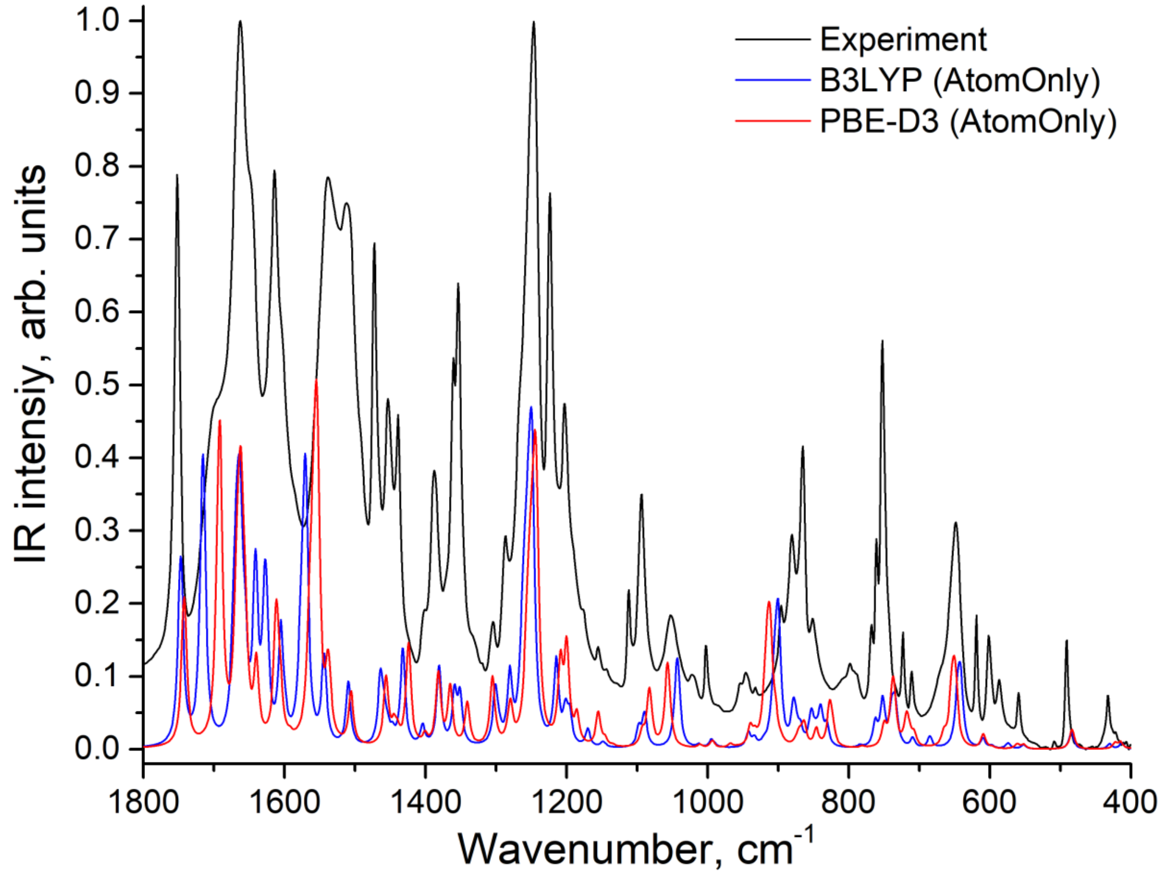

(b)

Figure S3. Experimental and theoretical IR spectra of [CRB+MLE] (1:1) in high-frequency (> 1800 cm^{-1}) (a) and mid-frequency (1800 ÷ 400 cm^{-1}) range (b)

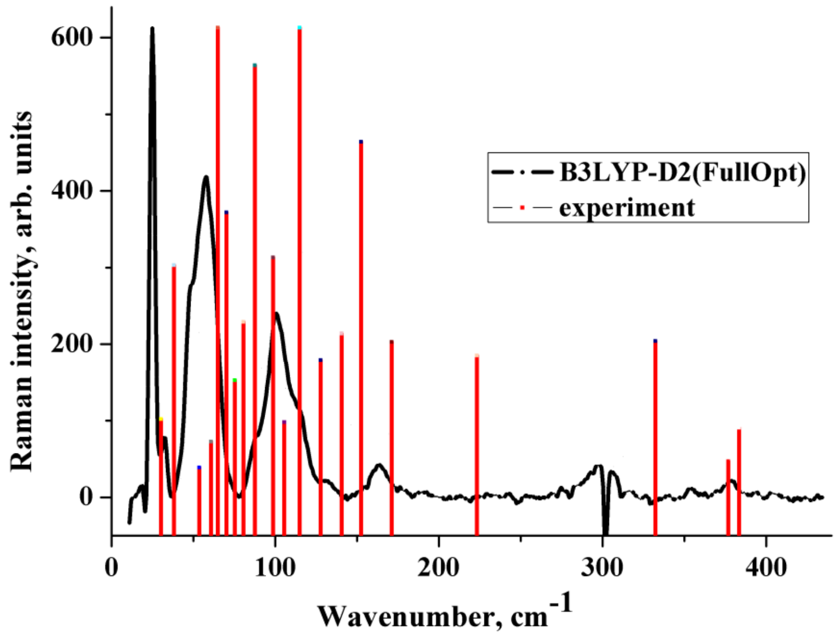

Figure S4. Raman spectrum of [CRB+MLE] (1:1). Experiment (black line) vs. B3LYP-D2(FullOpt) computations (red sticks). The height of the sticks is proportional to the relative Raman intensity of the corresponding transition.

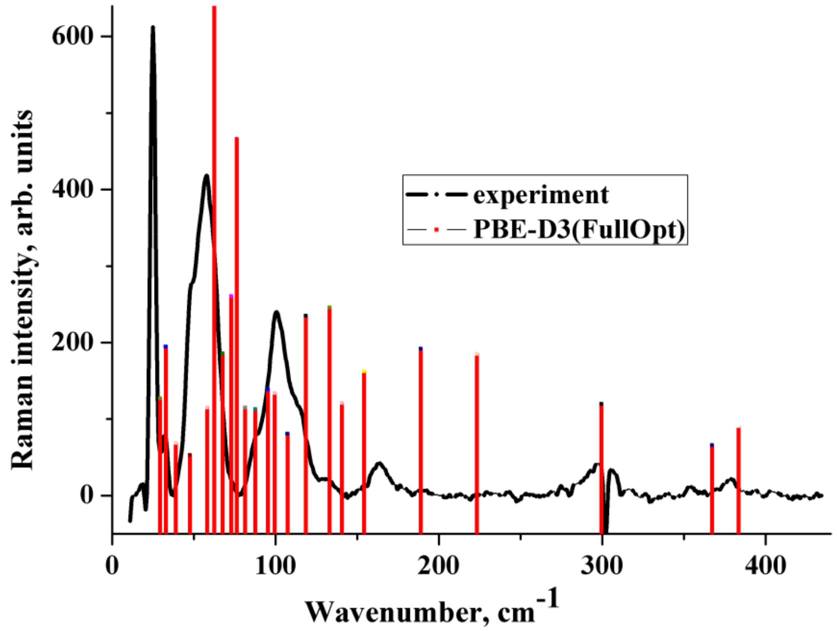

Figure S5. Raman spectrum of [CRB+MLE] (1:1). Experiment (black line) vs. PBE-D3(FullOpt) computations (red sticks). The height of the sticks is proportional to the relative Raman intensity of the corresponding transition.

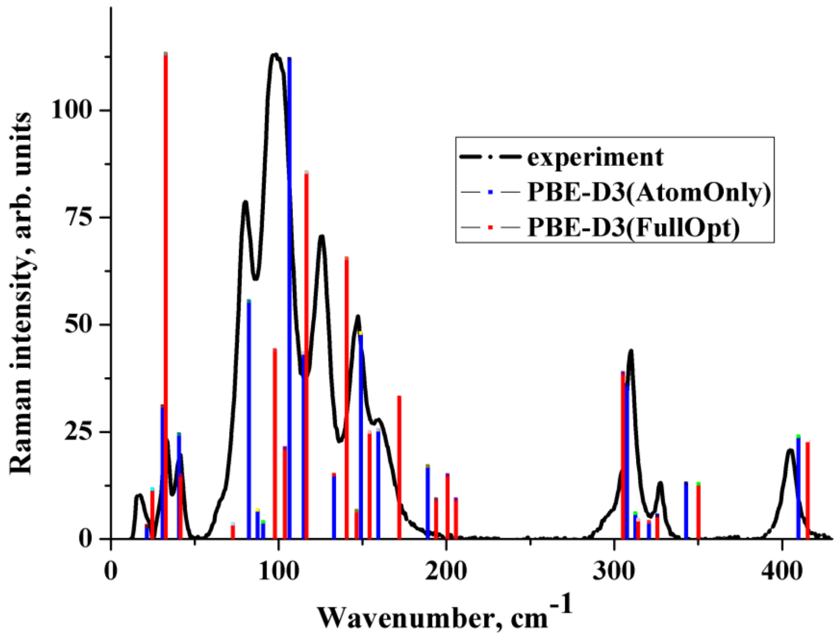

Figure S6. Raman spectrum of the crystalline maleic acid. Experiment (black line) vs. PBE-D3(AtomOnly) computations (blue sticks) and PBE-D3(FullOpt) computations (red sticks). The height of the sticks is proportional to the relative Raman intensity of the corresponding transition.

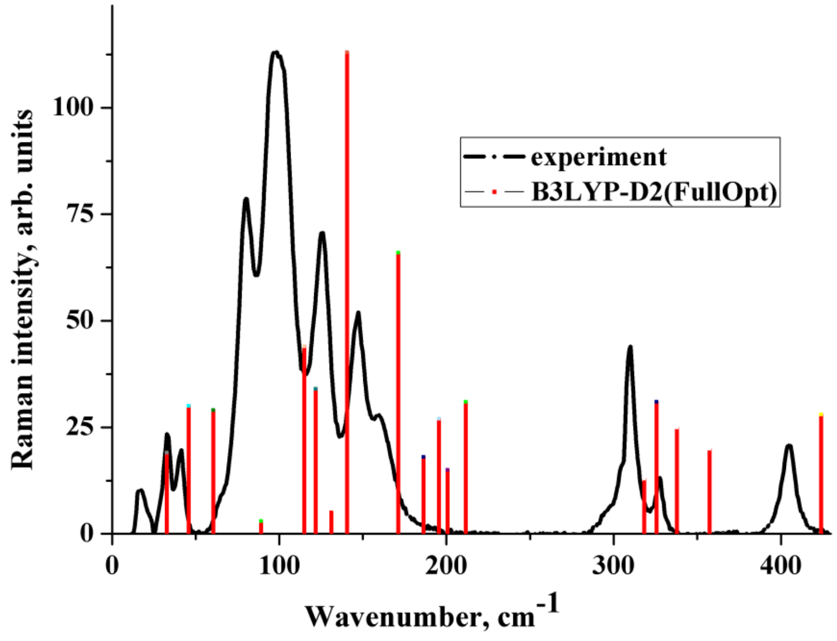

Figure S7. Raman spectrum of the crystalline maleic acid. Experiment (black line) vs. B3LYP-D2(FullOpt) computations (red sticks). The height of the sticks is proportional to the relative Raman intensity of the corresponding transition.

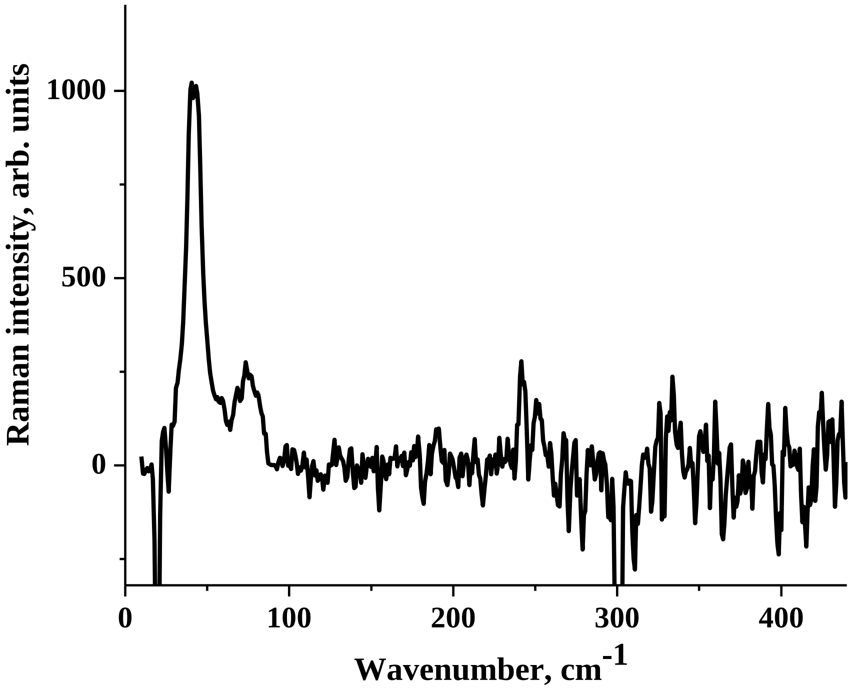

Figure S8. Raman spectrum of the CRB crystal. The dips in the spectrum at 20.2 cm^{-1} and at 302 cm^{-1} are artifacts of measurements associated with the presence of dust particles on the mirrors.

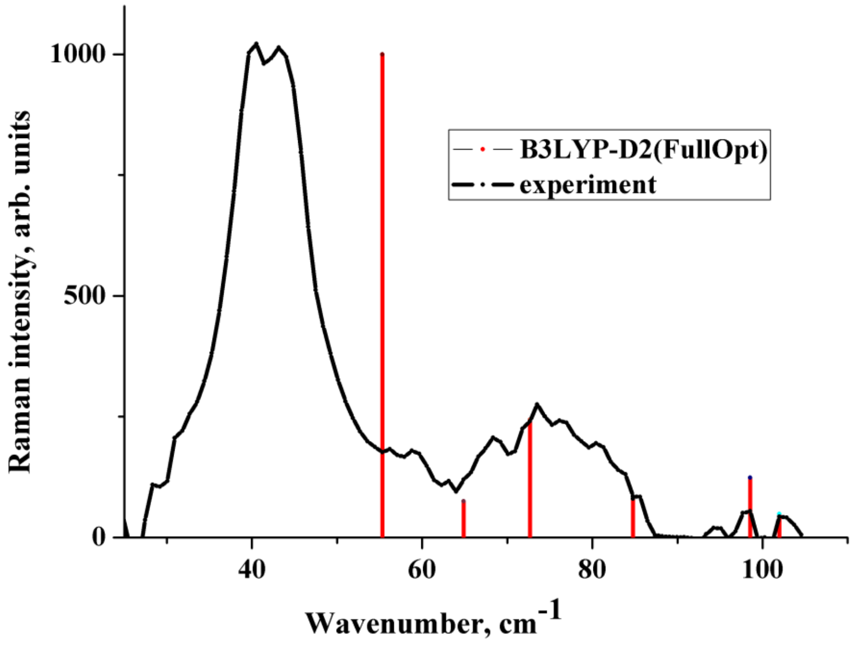

Figure S9. Raman spectrum of the of the CRB crystal in the THz region. Experiment (black line) vs. B3LYP-D2(FullOpt) computations (red sticks). The height of the sticks is proportional to the relative Raman intensity of the corresponding transition.

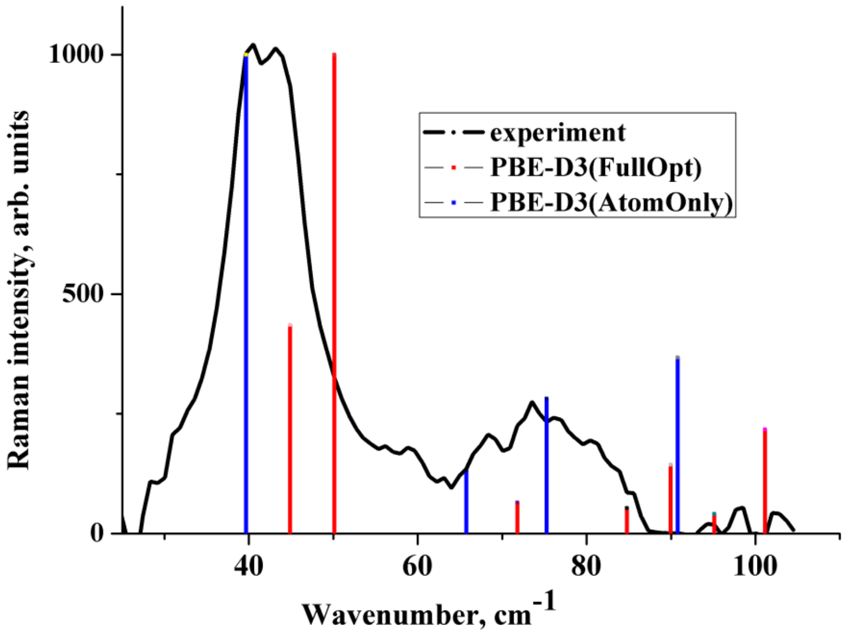

Figure S10. Raman spectrum of the of the CRB crystal in the THz region. Experiment (black line) vs. PBE-D3(AtomOnly) computations (blue sticks) and PBE-D3(FullOpt) computations (red sticks). The height of the sticks is proportional to the relative Raman intensity of the corresponding transition.

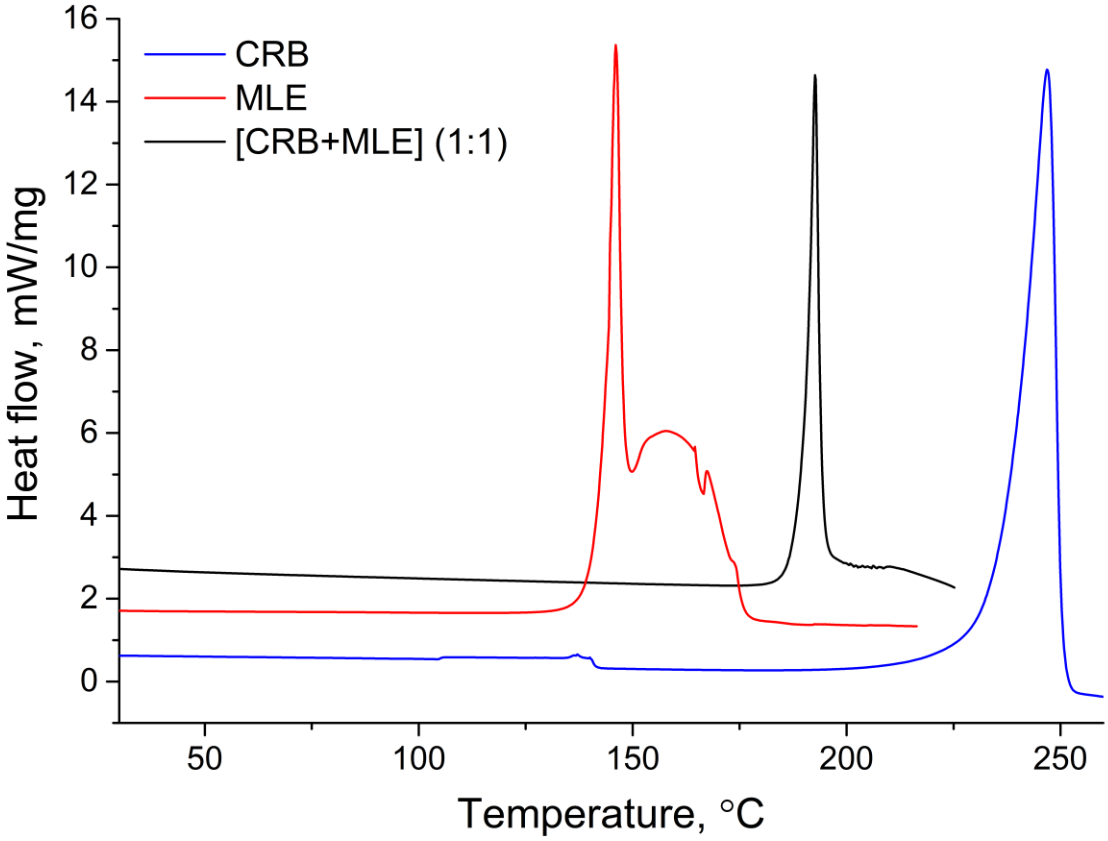

Figure S11. Results of DSC analysis of [CRB+MLE] (1:1) and its components in their pure forms.

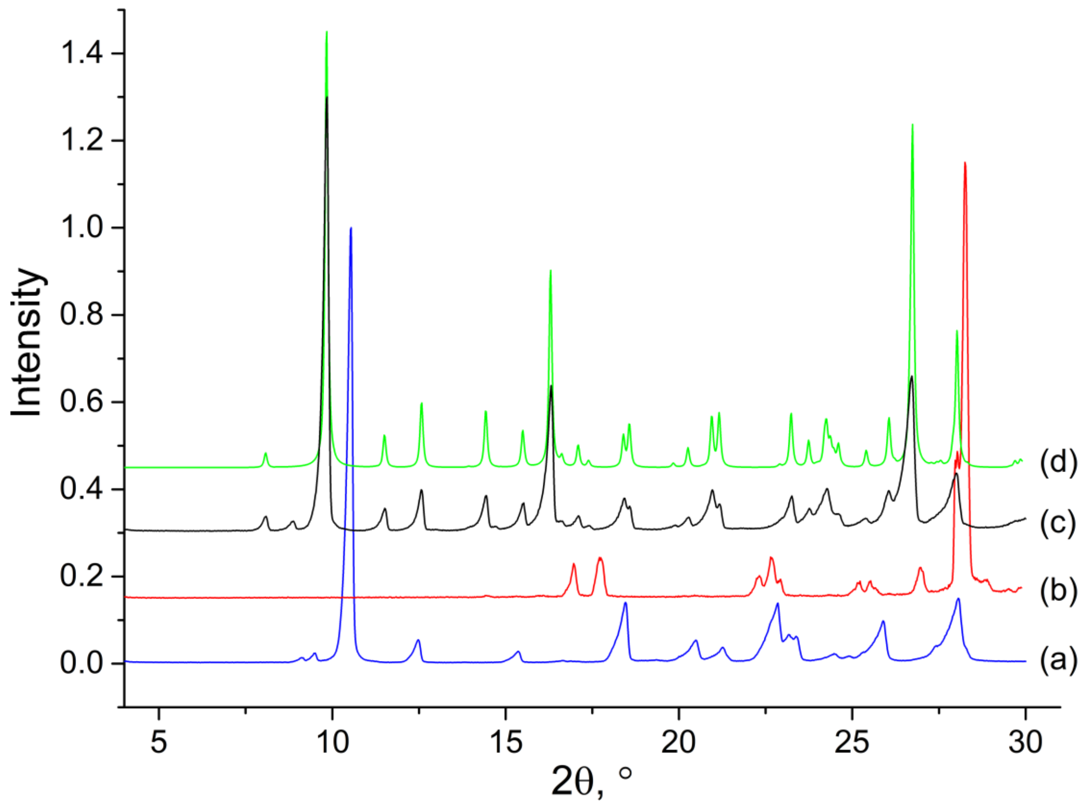

Figure S12. Powder X-Ray diffraction patterns for pure CRB (a), MLE (b), [CRB+MLE] (1:1) salt obtained by grinding with methanol (c) compared against the salt pattern simulated from the single crystal structure (d).

References

1. Dovesi, R.; Erba, A.; Orlando, R.; Zicovich-Wilson, C.M.; Civalleri, B.; Maschio, L.; Rérat, M.; Casassa, S.; Baima, J.; Salustro, S. Quantum-mechanical condensed matter simulations with CRYSTAL. *Wiley Interdisciplinary Reviews: Computational Molecular Science* **2018**, e1360.
2. Giannozzi, P.; Baroni, S.; Bonini, N.; Calandra, M.; Car, R.; Cavazzoni, C.; Ceresoli, D.; Chiarotti, G.L.; Cococcioni, M.; Dabo, I., et al. QUANTUM ESPRESSO: a modular and open-source software project for quantum simulations of materials. *Journal of Physics: Condensed Matter* **2009**, *21*, 395502, doi:10.1088/0953-8984/21/39/395502.
3. Giannozzi, P.; Andreussi, O.; Brumme, T.; Bunau, O.; Buongiorno Nardelli, M.; Calandra, M.; Car, R.; Cavazzoni, C.; Ceresoli, D.; Cococcioni, M., et al. Advanced capabilities for materials modelling with Quantum ESPRESSO. *Journal of Physics: Condensed Matter* **2017**, *29*, 465901, doi:10.1088/1361-648x/aa8f79.
4. Boys, S.F.; Bernardi, F. The calculation of small molecular interactions by the differences of separate total energies. Some procedures with reduced errors. *Molecular Physics* **1970**, *19*, 553-566, doi:10.1080/00268977000101561.
5. Gatti, C.; Saunders, V.R.; Roetti, C. Crystal field effects on the topological properties of the electron density in molecular crystals: The case of urea. **1994**, *101*, 10686-10696, doi:10.1063/1.467882.
6. Mata, I.; Alkorta, I.; Espinosa, E.; Molins, E. Relationships between interaction energy, intermolecular distance and electron density properties in hydrogen bonded complexes under external electric fields. *Chemical Physics Letters* **2011**, *507*, 185-189.
7. Vener, M.V.; Egorova, A.N.; Churakov, A.V.; Tsirelson, V.G. Intermolecular hydrogen bond energies in crystals evaluated using electron density properties: DFT computations with periodic boundary conditions. *Journal of computational chemistry* **2012**, *33*, 2303-2309, doi:10.1002/jcc.23062.
8. Shishkina, A.V.; Zhurov, V.V.; Stash, A.I.; Vener, M.V.; Pinkerton, A.A.; Tsirelson, V.G. Noncovalent interactions in crystalline picolinic acid N-oxide: insights from experimental and theoretical charge density analysis. *Crystal Growth Design* **2013**, *13*, 816-828.
9. Vener, M.V.; Shishkina, A.V.; Rykounov, A.A.; Tsirelson, V.G. Cl ···Cl interactions in molecular crystals: Insights from the theoretical charge density analysis. *The Journal of Physical Chemistry A* **2013**, *117*, 8459-8467.
10. Manin, A.N.; Voronin, A.P.; Manin, N.G.; Vener, M.V.; Shishkina, A.V.; Lermontov, A.S.; Perlovich, G.L. Salicylamide Cocrystals: Screening, Crystal Structure, Sublimation Thermodynamics, Dissolution, and Solid-State DFT Calculations. *The Journal of Physical Chemistry B* **2014**, *118*, 6803-6814, doi:10.1021/jp5032898.
11. Surov, A.O.; Churakov, A.V.; Perlovich, G.L. Three Polymorphic Forms of Ciprofloxacin Maleate: Formation Pathways, Crystal Structures, Calculations, and Thermodynamic Stability Aspects. *Crystal Growth Design* **2016**, *16*, 6556-6567.
12. Voronin, A.P.; Volkova, T.V.; Ilyukhin, A.B.; Trofimova, T.P.; Perlovich, G.L. Structural and energetic aspects of adamantane and memantine derivatives of sulfonamide molecular crystals: experimental and theoretical characterisation. *CrystEngComm* **2018**, *20*, 3476-3489, doi:10.1039/C8CE00426A.
13. Rozenberg, M.; Loewenschuss, A.; Marcus, Y. An empirical correlation between stretching vibration redshift and hydrogen bond length. *Physical Chemistry Chemical Physics* **2000**, *2*, 2699-2702, doi:10.1039/B002216K.
14. Mayo, S.L.; Olafson, B.D.; Goddard, W.A. DREIDING: a generic force field for molecular simulations. *The Journal of Physical Chemistry* **1990**, *94*, 8897-8909, doi:10.1021/j100389a010.
15. Blöchl, P.E. Projector augmented-wave method. *Physical Review B* **1994**, *50*, 17953-17979, doi:10.1103/PhysRevB.50.17953.
